# Retinal Vascular Degeneration in the Transgenic P23H Rat Model of Retinitis Pigmentosa

**DOI:** 10.3389/fnana.2018.00055

**Published:** 2018-06-29

**Authors:** Laura Fernández-Sánchez, Gema Esquiva, Isabel Pinilla, Pedro Lax, Nicolás Cuenca

**Affiliations:** ^1^Department of Optics, Pharmacology and Anatomy, University of Alicante, Alicante, Spain; ^2^Department of Physiology, Genetics and Microbiology, University of Alicante, Alicante, Spain; ^3^Department of Ophthalmology, Lozano Blesa University Hospital, Zaragoza, Spain; ^4^Aragon Institute for Health Research (IIS Aragon), Zaragoza, Spain; ^5^Institute Ramón Margalef, University of Alicante, Alicante, Spain

**Keywords:** vascular retinal network, retinal degeneration, retinitis pigmentosa, confocal microscopy, camera lucida, NADPH diaphorase

## Abstract

Retinitis pigmentosa (RP) is a group of inherited retinal degenerative diseases involving a progressive degeneration of photoreceptor cells. Following the loss of photoreceptors, retinal vascularization tends to decrease, which seems to play a role in the degenerative process of retinal cells. This study reports changes in retinal vascular network architecture in the P23H rat model of RP at different stages of retinal degeneration. Homozygous P23H line-3 rats of ages ranging from 18 days to 16 months were used in this study. Age-matched Sprague-Dawley (SD) rats were used as control animals. Vertical sections and wholemount retinas were immunolabeled for type IV collagen or stained using NADPH diaphorase histochemistry, and retinal vascular networks were drawn using a camera lucida. The superficial and deep capillary plexus (DCP) were fully developed at P18 in P23H rat retinas and showed no differences from the control animals. In 4-month-old P23H rat retinas, the superficial and intermediate capillary plexus were similar to those observed in age-matched SD rats, but a reduction in the DCP could be observed in these animals, with a significant decrease in both capillary density and capillary loops. At 16 months, the DCP was completely lost, and only vessels exhibiting an abnormal, tortuous dead-end could be observed. The middle capillary plexus had virtually disappeared at this age. Only perpendicular vessels connecting the superficial and DCP were found. The superficial plexus showed no changes in the vascular surface with age. In RP, photoreceptor loss is accompanied by degenerative changes in the retinal vascular network. The disruption of the capillary plexus, with loss of capillary density and capillary loops, can hamper the normal supply of oxygen and nutrients to retinal cells, thus accelerating retinal degeneration. Therefore, changes in retinal vascularization must be taken into account in the design of therapies targeting retinal degenerative diseases.

## Introduction

Retinitis pigmentosa (RP) constitutes a heterogeneous group of inherited neurodegenerative disorders of the retina characterized by progressive photoreceptor cell death and vision loss, which finally lead to blindness. Of all autosomal dominant RP (adRP) cases in humans, it is estimated that more than 25% are related to mutations in the rhodopsin-encoding gene (Hartong et al., 2006). One of the most common rhodopsin mutations is the P23H, which accounts for about one third of all cases of RP linked to the rhodopsin gene in the USA (Dryja et al., 2000).

Retinal neurodegenerative diseases involve numerous morphological and functional changes that cause a structural remodeling of retinal tissue (Jones et al., 2003; Marc et al., 2003; Cuenca et al., 2014). Among these changes, vascular alterations have been described. In RP patients, blood-retinal barrier (BRB) breakdown (Newsome, 1986), vascular attenuation (Milam et al., 1998b) and oxygen saturation alterations in retinal vessels (Eysteinsson et al., 2014; Türksever et al., 2014; Battu et al., 2015) have also been reported.

An adequate balance between oxygen supply and consumption is critical for retinal homeostasis (Yu et al., 2000; Yu and Cringle, 2005), and alterations in this balance play a determining role in the pathogenesis of many retinal diseases (Kaur et al., 2008; Pournaras et al., 2008; Cuenca et al., 2014). A disturbance of the retinal vascular pattern results in alterations in the blood supply that affect the oxygen delivery and metabolic substrates (Yu and Cringle, 2001, 2005). In this sense, increased O_2_ tension in the outer retina (Yu and Cringle, 2005) and a diminished number of vessel profiles in the deep capillary plexus (DCP) (Pennesi et al., 2008) have been described in animal models of RP. In addition, different authors have reported a migration of retinal pigment epithelial (RPE) cells along blood vessels as part of retinal remodeling in later stages of degeneration (Villegas-Pérez et al., 1998; Garcia-Ayuso et al., 2011).

Development of the retinal vasculature is mediated by neuroglia. Hypoxia-induced expression of vascular endothelial growth factor (VEGF), first by astrocytes and then by Müller cells, promotes the development of a superficial vascular plexus (SP) overlying the nerve fiber layer (NFL), followed by the formation of a DCP at the outer plexiform layer (OPL), and the belated emergence of a smaller intermediate retinal capillary plexus (ICP), located in the inner margin of the inner nuclear layer (INL) and connecting both superficial and deep plexuses (Stone et al., 1995; Provis, 2001; Gariano and Gardner, 2005; Kur et al., 2012; Cuenca et al., 2014). Retinal diseases induce reactive changes in glial cells (Bringmann et al., 2006; Coorey et al., 2012; Fernández-Sánchez et al., 2015a), which, in turn, result in an imbalance of pro-angiogenic and anti-angiogenic factors (Gariano and Gardner, 2005; Penn et al., 2008; Cuenca et al., 2014). In this context, it has been reported that the loss of photoreceptors during vascular development in rodent models of RP has a major impact on the number of capillary profiles in the DCP (Pennesi et al., 2008; Fernández-Sánchez et al., 2012a), and relatively little effect on the structure of the SP (Pennesi et al., 2008). Many other studies, however, have reported major alterations in both the superficial and DCP in different rodent models of RP, including the development in both plexuses of vascular anomalous complexes, which are produced as the result of the condensation of vessels associated with RPE cells that migrate into the neural retina (Villegas-Pérez et al., 1998; Wang et al., 2000; Fernández-Sánchez et al., 2015a; Pinilla et al., 2016).

The aim of this study was to examine morphological changes in the retinal vascular network and quantify modifications in both the superficial and deep retinal capillary plexuses during retinal degeneration in P23H line-3 rats, an animal model of adRP.

## Materials and Methods

### Animals

A total of 20 homozygous albino P23H line-3 rats received from Dr. M. LaVail (UCSF) were used at postnatal days 18–480 (P18–480) as a model of RP. Normal Sprague-Dawley (SD) rats (*n* = 16) provided by Harlan Laboratories (Barcelona, Spain) were used as age-matched controls. All rats originated from a colony bred in the animal facilities at the University of Alicante. Rats were maintained under controlled temperature and light conditions on a 12-h light/dark cycle. Dry food and water were provided *ad libitum*. The animals were housed, handled and the procedures carried out as specified in Project License UA-2013-07-22, which was approved by the Ethical Committee for Animal Experimentation at the University of Alicante. All procedures were carried out in accordance with current regulations on the care and use of laboratory animals (NIH-guidelines, EU Directive 2010/63/EU for animal experiments, and the ARVO Statement for the Use of Animals in Ophthalmic and Vision Research), minimizing animal numbers and suffering during the experiments.

### Retinal Histology

Animals were euthanized with an overdose of sodium pentobarbital (50 mg/kg i.p.). Prior to enucleation, a suture was sewn on the dorsal limbus of each eye to aid with orientation. A puncture was then made at the sclerocorneal limbus to allow the fixative solution to penetrate. The eyeballs were immersed in 4% (w/v) paraformaldehyde for 1 h, washed three times in phosphate-buffered saline (PB) and cryoprotected in 15%, 20% and 30% sucrose. The cornea, lens and vitreous body were removed, and the eyecups were processed for cryostat sections or wholemounts. For wholemount preparations, the retinas were cut into four portions (superior, inferior, nasal and temporal), spread out and flattened (ganglion-cell-side up). For cryostat sections, the eyecups were frozen in OCT with liquid nitrogen. Fourteen-micrometer-thick sections were obtained using a Leica CM 1900 cryostat (Leica Microsystems, Wetzlar, Germany), mounted on Superfrost Plus slides (Menzel GmbH & Co KG, Braunschweig, Germany) and air-dried.

### Retinal Immunohistochemistry

Immunofluorescence staining was performed on retinal sections or wholemount preparations in order to visualize the retinal vasculature. Staining was performed following standard protocols as previously described (Fernández-Sánchez et al., 2015a). Briefly, to improve the tissue permeability to the antibodies, wholemount retinas were incubated in 0.02% sodium borohydride (#163314; Panreac, Barcelona, Spain) in PB (5 min, RT). Sections and wholemount retinas were incubated with goat anti-collagen type IV antibodies (1:100; Millipore Cat # AB769) at 4°C temperature overnight or for 3 days, respectively. After washing three times for 10 min in PB, the retinas were incubated for 1 h or overnight, respectively, in Alexa Fluor 488 (green)-conjugated anti-goat IgG from Molecular Probes (Eugene, OR, USA) at a dilution rate of 1:100. Finally, the preparations were washed in PB, mounted in Citifluor (Citifluor Ltd., UK) and coverslipped for viewing under fluorescence microscopy, using a Leica CTR MIC microscope (Leica Microsystems, Germany).

### NADPH-Diaphorase Histochemistry

Histochemistry of reduced nicotinamide adenine dinucleotide phosphate diaphorase (NADPH-d) was performed on wholemount retinas to visualize the retinal vascular network, as already described (Haverkamp et al., 2000; Fernández-Sánchez et al., 2012a). In brief, the retinas were incubated at 37°C for 2 h in PB containing 1 mg/ml NADPH (Sigma, St. Quentin Fallavier, France), 0.1 mg/ml nitroblue tetrazolium (NBT) (Sigma, St. Quentin Fallavier, France) and 1% (v/v) Triton X-100. Afterwards, the retinas were washed three times in PB and mounted in Citifluor mounting medium (Citifluor Ltd.).

### Vascular Network Quantification

Both the superficial and the deep vascular plexuses from wholemount retinas stained with NADPH-d were drawn with the aid of a camera lucida coupled to a Leica DMR microscope (Leica Microsystems). Since the NADPH-d staining allows arteries to be distinguished from veins, arteries and veins from the SP were drawn in red and blue, respectively. The drawings were digitalized and the resulting images were adjusted for equal brightness and contrast using Adobe Photoshop software (Adobe Systems Inc., San Jose, CA, USA). The morphometric analysis was performed with the aid of ImageJ software (National Institutes of Health, Bethesda, MD, USA). The relative capillary density of both DCP and SP were measured using the ImageJ function “Measure Area” and expressed as the ratio between the total blood vessel area and the retinal surface. In the images from the SP, arteries and veins were analyzed separately. For the structural study of capillary loops, images were analyzed using the “Analyze Particles” function of the ImageJ software. Capillary loop density, expressed as the number of capillary loops per retinal area, and capillary loop area, expressed as the area circumscribed within visually enclosed capillary loops, were measured. By using size and circularity cut-off ranges, we selected which particles to analyze, in order to include only identifiable capillary loops in the test. The area of the capillary loops included in the analysis ranged from 200 μm^2^ to 20,000 μm^2^. The ImageJ software tools also allowed the establishment of particle size ranges (1000 μm^2^ each from 200 μm^2^ to 20,000 μm^2^) and the quantification of the number of loops for each size range.

### Statistical Analysis

Statistical analyses were performed using Prism software (GraphPad Software, San Diego, CA, USA). A two-way ANOVA was calculated to assess the differences in genotype (SD vs. P23H) and age (P18–16 months), individually and in combination. If the level of significance was 0.05 or less, *post hoc* pairwise comparisons using Bonferroni’s test were carried out. Normal distribution and homogeneity of variance were calculated for the categories of the aforementioned variables. Data were plotted representing the mean ± standard error of the mean (SEM). *P* values of less than 0.05 were considered statistically significant.

## Results

### Morphological Alterations in the P23H Rat Retinal Vasculature

**Figure 1:**
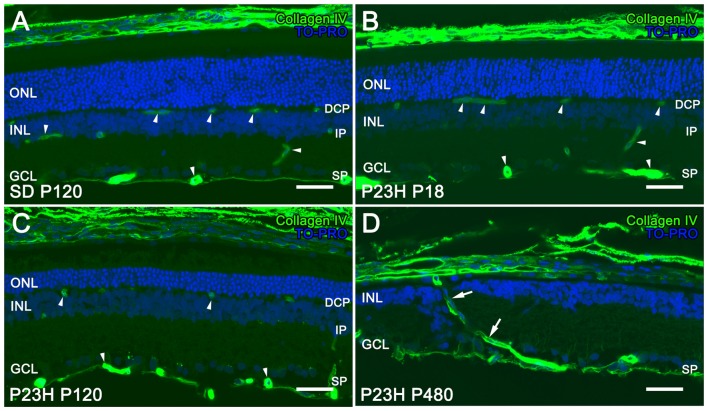
Vascular changes associated with photoreceptor loss in P23H rats. Immunolabeling of retinal vertical sections from a Sprague-Dawley (SD) rat at P120 **(A)** and P23H rats **(B–D)** at P18 **(B)**, P120 **(C)** and P480 **(D)** stained for collagen type IV (green). Nuclei stained with a nuclear marker (TO-PRO3, blue). All images were taken in the central area of the retina, close to the optic nerve. The loss of photoreceptor cells in P23H rats is accompanied by a progressive loss of capillary vessels (arrowheads), initially affecting the deep capillary plexus (DCP). At P480 the deeper and intermediate plexuses were virtually disappeared in the P23H rat retina, and choroidal vessels (arrows) invaded the retina displacing inner nuclear layer (INL) cells **(D)**. ONL: outer nuclear layer; INL: inner nuclear layer; GCL: ganglion cell layer; DCP: deep capillary plexus IP: intermediate plexus; SP: superficial plexus. Scale bar: 40 μm.

The retinal vasculature in rodents is distributed in a trilaminar vascular network formed by a superficial vascular plexus and a DCP, both connected by an intermediate layer of retinal vessels running along the inner edge of the INL (Figure 1A). In P23H rats, photoreceptor cell death and topological restructuring of the degenerative retina were accompanied by progressive age-related changes in the retinal vasculature. At P18, there were no noticeable differences in terms of retinal architecture between SD control and P23H rats (Figures 1A,B, respectively). However, in 4-month-old P23H rats photoreceptor loss was associated to a reduced vessel density in the DCP (Figure 1C). In advanced stages of degeneration (P480) the complete loss of rod and cone photoreceptors triggered subsequent topological restructuring of the retina (Figure 1D). The deeper and intermediate plexuses were virtually disappeared, and the superficial plexus showed noticeable histological abnormalities. RPE cells migrated into the retina, often with accompanying choroidal vessels, passing through gaps in the glial seal, and displacing INL cells (Figure 1D).

**Figure 2:**
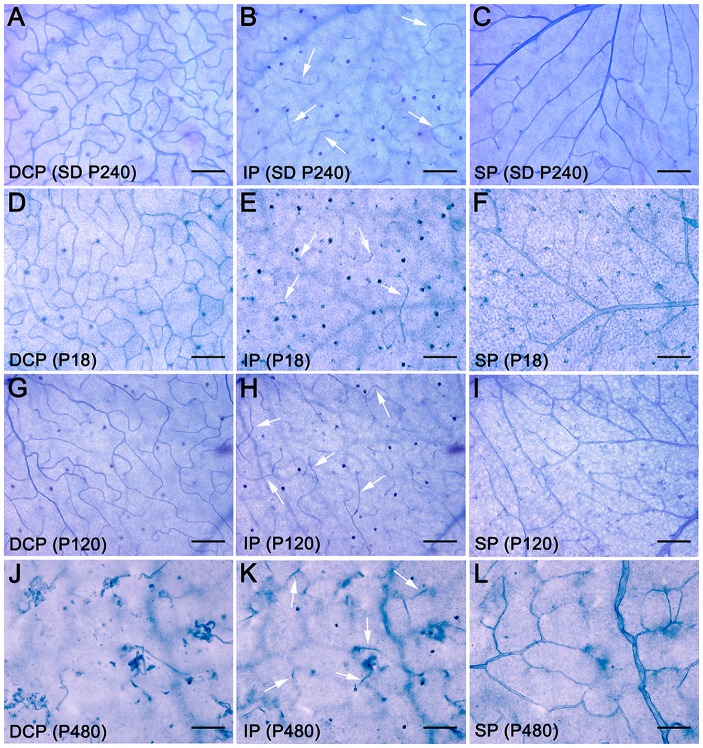
Age-related changes in the retinal vasculature in P23H rats. Wholemount retinas from a SD rat at P240 **(A–C)** and P23H rats at P18 **(D–F)**, P120 **(G–I)** and P480 **(J–L)** stained with NAPDH diaphorase, showing the DCP **(A,D,G,J)**, intermediate plexus (IP) **(B,E,H,K)**, arrows, and superficial vascular plexus (SP) **(C,F,I,L)**. All images were taken in the medial area of the retina, between the major blood vessels. During aging, the retinal vasculature of P23H rats shows early and rapid degeneration in the DCP, intermediate rate of degeneration in the IP, and slight delayed changes in the SP. Scale bar: 100 μm.

To better understand degenerative changes in the retinal vascular network of P23H rats, we analyzed the density, morphology and distribution of retinal vessels on wholemount retinas from normal and diseased animals. Figure 2 shows the three retinal vascular plexuses from P18, P120 and P480 P23H (Figures 2D–L) rats and from a P240 SD rat (Figures 2A–C), stained according to the NADPH-d method. The superficial and deep vascular plexuses were fully developed at P18 in P23H rat retinas, where it was possible to observe a mature DCP, with well-formed capillary loops (Figure 2D), and a complete SP, with well-differentiated arteries and veins (Figure 2F), as compared to wild-type SD retinas (Figures 2A,C, respectively). Conversely, the intermediate plexus of the retinal vasculature was incompletely developed in P18 P23H rats (Figure 2E), which showed only a few horizontally extending capillaries, as opposed to the continuous capillary network observed in SD rats (Figure 2B).

In the 4-month-old P23H retina (Figures 2G–I), a less dense capillary network was observed in the DCP, with a noticeable loss of retinal capillary loops (Figure 2G). Conversely, the intermediate and superficial vascular plexuses showed a normal structure (Figures 2H,I, respectively). At an age of 16 months, the DCP in the P23H rat retina was completely lost, and only isolated vessels exhibiting an abnormal, tortuous dead-end could be observed (Figure 2J). On the other hand, the intermediate plexus had virtually disappeared by this age, and the remaining vessels were positioned perpendicularly to the SP and DCP (Figure 2K). The superficial plexus at this age also showed noticeable histological abnormalities (Figure 2L).

**Figure 3:**
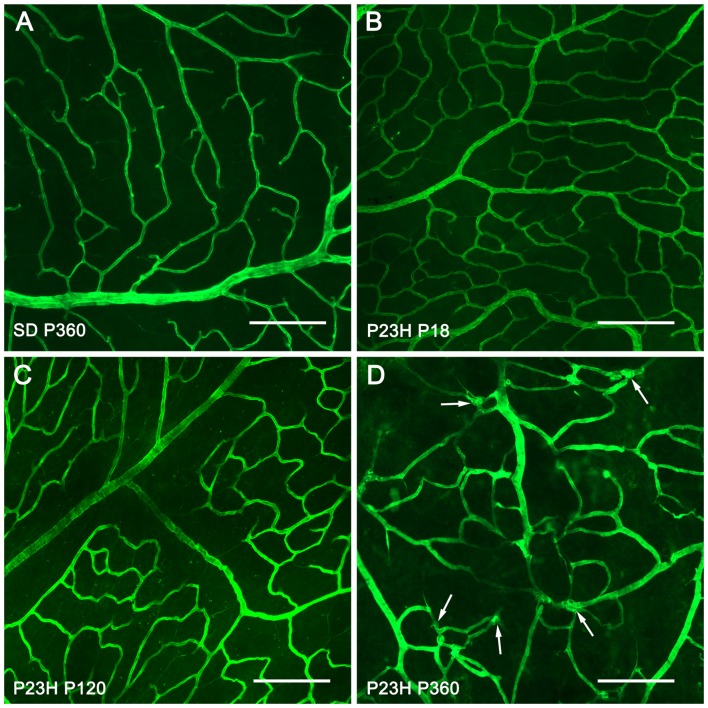
Morphological changes in the superficial retinal plexus in P23H rats. Wholemount retinas from a P360 SD rat **(A)** and P23H rats at P18 **(B)** P120 **(C)** and P360 **(D)** stained for collagen IV in order to show the blood vessels of the superficial retinal plexus during retinal degeneration. All images were taken in the central area of the retina, close to the optic nerve. In advanced stages of retinal degeneration (P360), the SP of P23H rats showed blood vessel tangles **(D)**, arrows; these alterations were not observed in age-matched control SD rats **(A)**. Scale bar: 200 μm.

For a better understanding of the morphological changes in the superficial retinal plexus of P23H rats, we performed immunofluorescence staining of wholemount retinas using antibodies against collagen type IV (Figure 3). Images showed normal retinal superficial plexuses at P18 and P120 in P23H rat retinas (Figures 3B,C), as compared to those observed in wild-type retinas (Figure 3A). At P360, as previously evidenced by NADPH-d techniques, the retinal superficial plexus showed morphological abnormalities, with tortuous vessels and a high number of vascular tangles (Figure 3D, arrows).

### Quantitative Analysis of the Retinal Vascular Network

**Figure 4:**
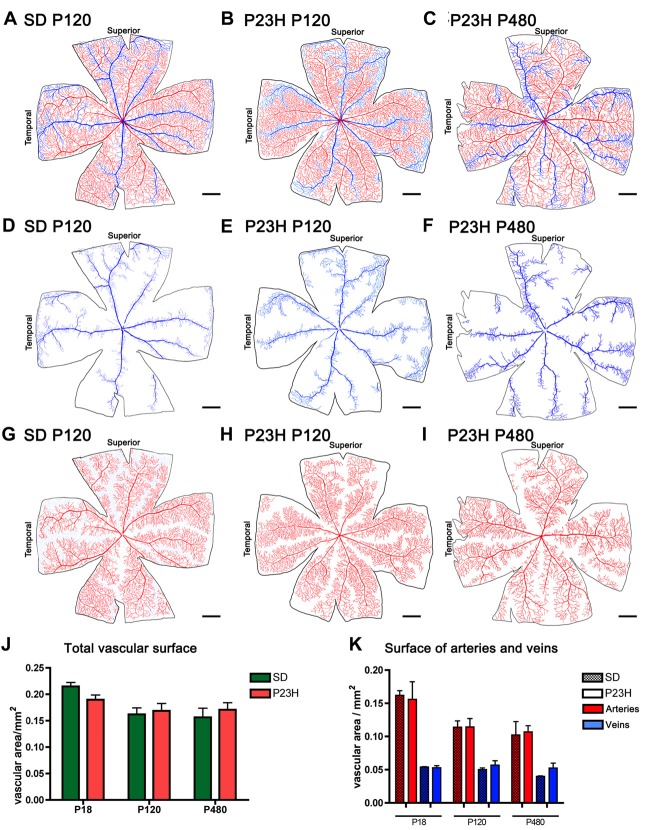
Surface area of the superficial vascular plexus in SD and P23H rats. **(A–I)** Representative drawings of the veins (blue) **(D–F)**, arteries (red) **(G–I)** or both **(A–C)** in the superficial retinal vasculature of P120 SD rats **(A,D,G)** and P23H rats at P120 **(B,E,H)** and P480 **(C,F,I)**. **(J,K)** Mean total vascular surface area **(J)** and mean surface area of arteries and veins **(K)** in SD and P23H rats at P18, P120 and P480. No differences in the total vascular surface area **(J)** or in the surface area of arteries and veins **(K)** were observed between P23H rats and age-matched control SD rats. Scale bar: 1 mm.

To better analyze degenerative changes in both the superficial and deep plexuses, blood vessels in wholemount retinas were stained with NADPH-d histochemistry, drawn using a camera lucida, and quantified with ImageJ software. The total surface area of the superficial vascular plexus (Figures 4A–C) and the surface area of veins (Figures 4D–F) and arteries (Figures 4G–I) were quantified separately. Despite the morphological alterations described above for the superficial vascular plexus of P23H rat retinas, no significant differences in the total vascular surface area (Figure 4J) nor in the surface area of arteries and veins (Figure 4K) were found between P23H rats at P18, P120 and P480 and age-matched wild-type retinas.

**Figure 5:**
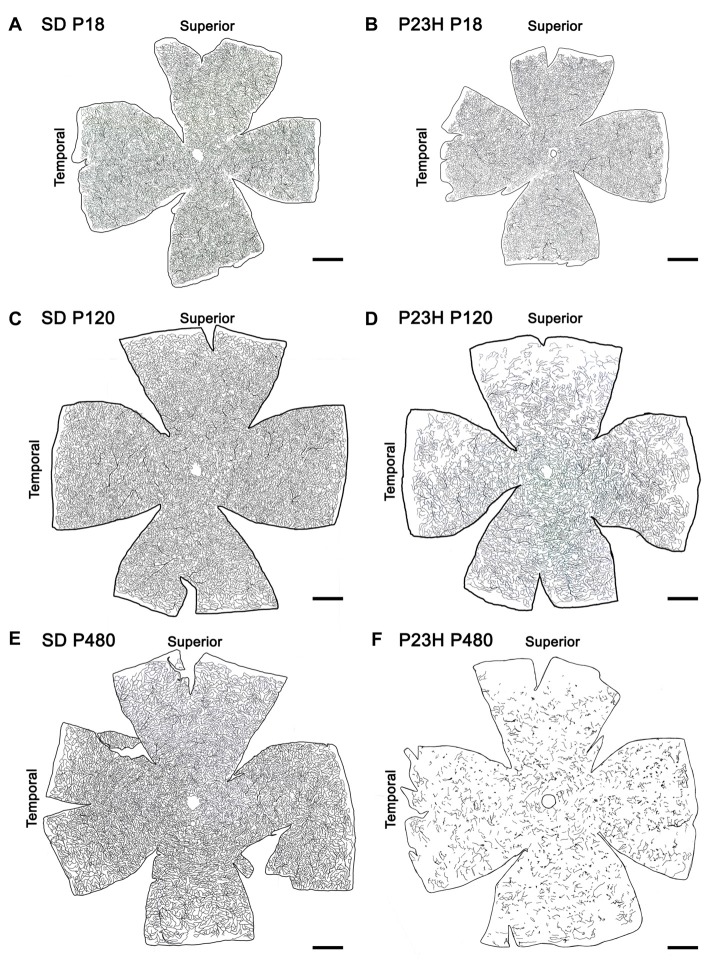
Age-related changes in the DCP in P23H rats. Representative drawings of the blood vessels in the DCP of SD **(A,C,E)** and P23H rats **(B,D,F)** at P18 **(A,B)**, P120 **(C,D)** and P480 **(E,F)**. P23H rats showed a progressive age-dependent regression in the deep retinal vasculature as compared to age-matched control SD rats. At P480, P23H rat retinas showed few scattered blood vessels **(F)**. Scale bar: 1 mm.

**Figure 6:**
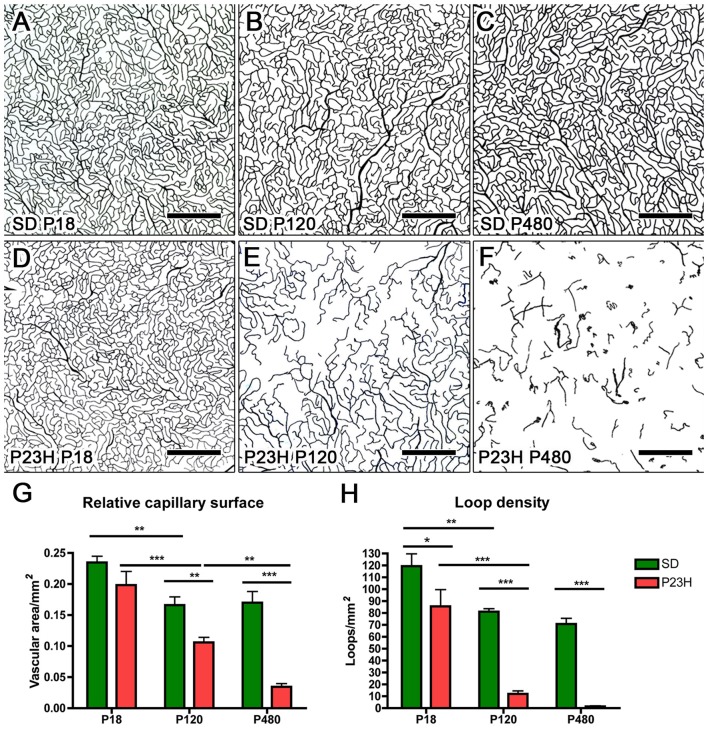
Quantitative changes in the DCP in P23H rats. **(A–F)** Representative drawings of the blood vessels in the DCP of SD **(A–C)** and P23H rats **(D–F)** at P18 **(A,D)**, P120 **(B,E)** and P480 **(C,F)**. All images were taken in the medial temporal retina. **(G)** Mean relative capillary density (capillary area/retinal area) in the entire DCP of both SD and P23H rat retinas, showing a progressive loss in the relative capillary surface area in P23H rats. **(H)** Mean number of capillary loops in the entire DCP of SD and P23H rat retinas, showing a progressive reduction in capillary loop density in P23H rats. *P < 0.05, **P < 0.01, ***P < 0.001; ANOVA, Bonferroni’s test. Scale bar: 500 μm.

Drawings of the retinal DCP from wild-type and P23H rats at P18, P120 and P480 (Figure 5) showed a progressive age-dependent reduction in the deep retinal vasculature of P23H rats (Figures 5B,D,F) as compared to that observed in wild-type SD rats (Figures 5A,C,E). At P120 the loss of capillary vessels was clearly noticeable along the peripheral retina of P23H rats (Figure 5D). At P480, P23H rat retinas showed only few scattered blood vessels (Figure 5F). Figures 6A–F shows higher magnification images of the retinal deep capillary vessels in a representative area of the temporal part of the retina in SD and P23H rats at P18, P120 and P480, evidencing the progressive age-dependent reduction of deeper retinal capillaries in P23H rats. Quantitative analysis of capillary density in the entire DCP of both SD and P23H rat retinas showed a progressive loss in the relative capillary surface area (vascular area/retinal area) of P23H rats from P18 to P480 (Figure 6G). More concretely, the DCP relative area in these animals was significantly (*P* < 0.01) smaller at P480 (0.03 ± 0.005, *n* = 3) than at P120 (0.11 ± 0.008, *n* = 5), and significantly (*P* < 0.001) smaller at P120 than at P18 (0.18 ± 0.017, *n* = 3). Moreover, the DCP relative area of P23H rats was significantly smaller than that of SD rats at P120 and P480 (*P* < 0.01 and *P* < 0.001, respectively). The DCP relative area of SD rats significantly decreased (*P* < 0.01) from P18 (0.23 ± 0.01, *n* = 4) to P120 (0.17 ± 0.013, *n* = 6). This result could be explained by the relative increase (around 30%) in the total retinal area observed in this period (Figures 5A,C). No changes in the DCP relative area were observed in SD rat retinas between P120 and P480 (0.17 ± 0.018, *n* = 5).

### Quantification of Capillary Loops in the DCP

In addition to capillary area, the density of capillary loops was quantified throughout the entire DCP (Figure 6H). As it was the case for the relative capillary surface, the mean density of capillary loops in the DCP progressively decreased in P23H rats. In these animals, the mean density of capillary loops measured at P120 (12.0 ± 2.45 loops/mm^2^, *n* = 5) and P480 (1.4 ± 0.45 loops/mm^2^, *n* = 3) was significantly (*P* < 0.001 in both cases) lower than that obtained at P18 (85.6 ± 10.26 loops/mm^2^, *n* = 3) (Figure 6H). Moreover, mean loop density in P23H rats at P18, P120 and P480 was significantly (*P* < 0.05 for P18 and *P* < 0.001 for P120 and P480) smaller than that of SD rats. In SD rats, the mean density of capillary loops significantly decreased between P18 (119.3 ± 10.42 loops/mm^2^, *n* = 4) and P120 (81.1 ± 2.44 loops/mm^2^, *n* = 5), but this difference can be explained, at least in part, by the above-mentioned increase in the total retinal area.

**Figure 7:**
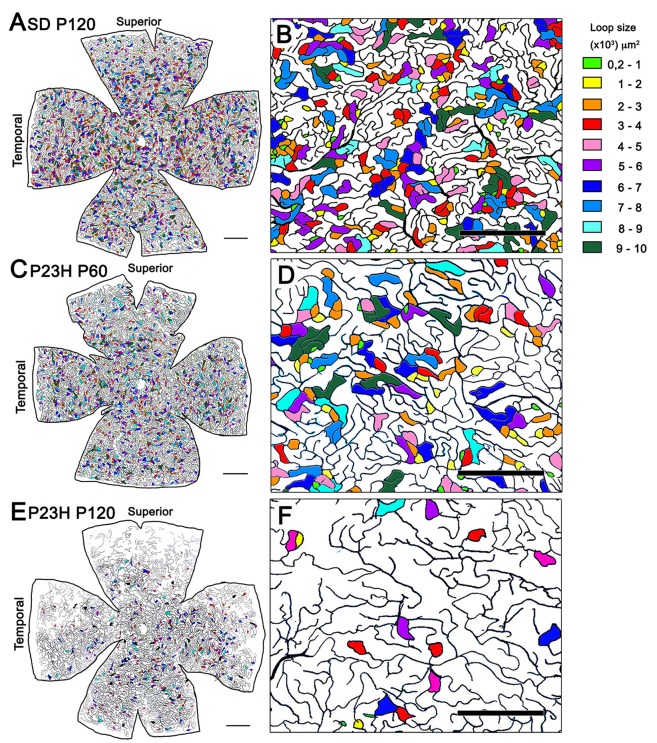
Capillary loop size and distribution in the DCP in P23H rats. **(A,C,E)** Representative drawings of the blood vessels in the DCP of a P120 SD rat **(A)** and P23H rats at P60 **(C)** and P120 **(E)** showing capillary loop size ranges from 200 μm^2^ to 10,000 μm^2^. To better visualize the relationship between capillary loop size and distribution, the area circumscribed within each loop has been labeled in different colors depending on their relative size. **(B,D,F)** Higher magnifications of the panels **(A,C,E)** respectively, corresponding to representative areas of the medial superior retina. Units on the right of the figure indicate the loop size ranges in μm^2^. Note the progressive reduction in capillary loop density in P23H rats. Scale bar: 1 mm for **(A,C)** and **(E)**; 500 μm for **(B,D,F)**.

To analyze the changes in the vascular pattern of the DCP in greater detail, the area circumscribed within each visually enclosed capillary loop was measured throughout the entire retina of P23H rats during the first states of retinal degeneration (P18, P60 and P120) and in age-matched SD rats. As we can see in Figure 7, the capillary loop size was heterogeneous in both SD and P23H rat retinas, with capillary loop areas ranging from 200 μm^2^ to more than 10,000 μm^2^. In all cases, both large and small loops were homogeneously distributed across the entire retina. In P23H rats, we evidenced a progressive reduction in capillary loop density. The loss of capillary loops was already evident at P60, with greater loss of loops in the peripheral retina (Figures 7C,D), as compared to that observed in wild-type control animals (Figures 7A,B). In P120 P23H rat retinas, the few capillary loops observed were located in the medial and central retina, mostly arranged in the inferior and nasal parts of the retina (Figures 7E,F).

**Figure 8:**
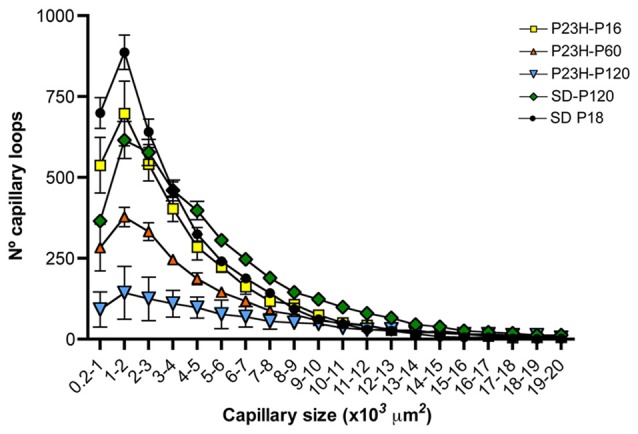
Relationship between capillary loop size and density in the DCP of P23H rats. Mean number of capillary loops for each of the 20 size ranges (from 200 μm^2^ to 20,000 μm^2^). Note that the relative loss of capillary loops in P23H rats was inversely proportional to their size.

The particle size distribution analysis showed that, regardless of the animal strain (SD or P23H) or age (P18, P60 or P120), the number of capillary loops in the DCP declines exponentially (f = a*exp(−b*x), *P* < 0.0001 in all cases) with respect to its size (Figure 8). The number of small capillary loops was therefore larger than that of large capillary loops. In P18 P23H rats, the particle size distribution showed similarities to that observed in SD rats at both P18 and P120 (Figure 8). However, a progressive age-dependent loss of the smaller capillary loops was observed in P60 and P120 P23H rats. The relative loss of capillary loops was inversely proportional to the loop size (f = y0+a*x, *P* < 0.001 in all cases).

## Discussion

The present study shows evidence that photoreceptor loss is accompanied by degenerative changes in all the three retinal vascular layers: superficial, intermediate and the DCP. Previous studies have demonstrated vascular alterations in RP animal models (Villegas-Pérez et al., 1998; Penn et al., 2000; Pennesi et al., 2008; Fernández-Sánchez et al., 2012a; Lopez Torres et al., 2015; Liu et al., 2016) and patients (Newsome, 1986; Milam et al., 1998a; Eysteinsson et al., 2014; Türksever et al., 2014; Battu et al., 2015). In this work, we describe in depth the vascular degenerative process in the P23H transgenic rat model of RP during the progression of the disease and in age-matched healthy retinas.

The transgenic P23H rat has been designed to reproduce the clinical signs observed in P23H RP patients (Steinberg et al., 1996; Machida et al., 2000). The gradual photoreceptor loss characteristic of P23H line-3 rats provides an experimental model closer to RP patients than other animal models (Machida et al., 2000; Cuenca et al., 2004). This vascular study completes a series of studies conducted by our research group analyzing the morphological and functional changes occurring during the degenerative process of homozygous P23H line-3 rats, including changes in astrocytes, microglia and Müller cells (Esquiva et al., 2017; Cuenca et al., 2014; Fernández-Sánchez et al., 2015a; Lax et al., 2016; Noailles et al., 2016, 2018). We have chosen homozygous vs. heterozygous P23H line-3 rats because our previous results have demonstrated that degeneration in the homozygous model is more aggressive but slow enough to properly assess neuroprotective effects of various compounds (Fernández-Sánchez et al., 2011, 2012a,b, 2015b, 2017; Lax et al., 2011; Noailles et al., 2014). On the other hand, this animal model allows to assess changes in retinal vasculature in both the initial and late severe stages of retinal degeneration in experiments not longer than 16–18 month (Lax et al., 2011; Esquiva et al., 2017; Fernández-Sánchez et al., 2015a). In our results, the late onset of retinal degeneration in P23H line-3 rats allowed for a normal development of the retinal vasculature. At P18, the superficial and deep vascular plexuses of P23H line-3 rats were fully developed, which is in agreement with what has previously been reported for transgenic animal lines with late and slow photoreceptor loss (Pennesi et al., 2008). Conversely, the intermediate plexus of the retinal vasculature was incompletely developed in P18 P23H rats, in accordance with previous studies showing that the intermediate vascular plexus in rodents is formed during the third postnatal week (Dorrell et al., 2002) and correlated temporally with the timing of synaptogenesis and amacrine cells maturation (Usui et al., 2015).

In adult dystrophic rats, a progressive age-dependent loss of deep capillary vessels was observed. This is in agreement with earlier studies that report profound retinal capillary atrophy associated with the death of photoreceptor cells (Penn et al., 2000; Wang et al., 2003; Pennesi et al., 2008; Fernández-Sánchez et al., 2012a). In our results, the loss of capillaries in the DCP begins in the peripheral retina and gradually progresses toward the more central retina. This pattern of vascular degeneration is consistent with the pattern of photoreceptor degeneration in RP. Previous studies in animal models for RP and in human RP retinas report that rod death typically starts in the mid peripheral retina and spreads over time to affect the macula and the more peripheral retina (Milam et al., 1998a; Fernández-Sánchez et al., 2011; Lax et al., 2014).

Additionally to the loss of capillary vessels, our results showed a progressive loss of capillary loops in the DCP of P23H rats. As in the vessels, the loop degeneration began in the peripheral retina and progressively spread toward the more central retina. At advanced stages of photoreceptor loss, the few capillary loops observed were arranged in the inferior and nasal parts of the central retina, the areas where most photoreceptors are left during the degenerative process (Fernández-Sánchez et al., 2011; Lax et al., 2014). Moreover, the progressive loss of capillary loops in P23H rat retinas was inversely proportional to the loop size, in other words, primarily the small capillary loops disappeared. This fact involved a progressive increase in the mean retinal surface perfused by each remaining capillary loop. The area bounded by retinal capillary loops has previously been quantified by others (Arend et al., 1991; Chan et al., 2012), but, to our knowledge, this is the first study in which the area of each capillary loop and the number of loops for each size range has been measured in the DCP as a suitable tool for evaluating retinal vascular degeneration. Our results show a correlation between the loss of capillary surface and the loss of loop density in the DCP of P23H rats. However, the decrease in the number of loops was more pronounced than the reduction of capillary surface. This suggests that the measurement in the number of loops may be a more sensitive indicator of vascular degeneration than the measurement of the vascular surface. Previous studies have proved that degeneration of retinal capillaries (vessels having no nuclei) is appreciable in early stages of retinal vascular degeneration (Liu et al., 2016). Here we show that decreased capillary loop density, detectable when photoreceptor cells are still largely present, also represents an early event in retinal vascular degeneration.

Photoreceptor cell death is concomitant to degeneration in the inner retina (Marc et al., 2003; Cuenca et al., 2004, 2014; Puthussery and Taylor, 2010; Jones et al., 2016), and results in progressive loss of retinal ganglion cells (García-Ayuso et al., 2010; Kolomiets et al., 2010) and decreased density of melanopsin-containing ganglion cells in late stages of retinal degeneration (Esquiva et al., 2017; Garcìa-Ayuso et al., 2015; Lax et al., 2016). Moreover, a previous study from our research group demonstrated that outer retinal degeneration induces astrogliosis and Müller cell activation (Fernández-Sánchez et al., 2015a). Here we show histological alterations in the structure of the inner retinal vascular plexus at advanced stages of outer retinal dystrophy. On the one hand, the superficial plexus from diseased retinas exhibited tortuous vessels and a high number of vascular tangles. This is in agreement with previous studies showing tortuous vessels in the superficial plexus of diseased retinas (Semkova et al., 2002; Flynn and Chan-Ling, 2006), including RP retinas (Wang et al., 2003; Fernández-Sánchez et al., 2015a), and in the superficial plexus of aged retinas (Hughes et al., 2006). Capillary tangles have also been previously described in the superficial plexus of degenerative retinas (Flynn and Chan-Ling, 2006; Fernández-Sánchez et al., 2015a).

Despite all these changes in the structure of the inner retinal vasculature, no significant changes in the total surface area of the superficial vascular plexus or in the surface area of retinal arteries and veins were found between RP rats and age-matched control rats, even in late stages of retinal degeneration. These results agree with those found in previous studies, which showed relative sparing of the superficial plexus in RP animals (Penn et al., 2000; Pennesi et al., 2008; Fernández-Sánchez et al., 2015a) and patients (Battaglia Parodi et al., 2017). A recent study in humans has demonstrated a significant reduction in superficial capillary plexus density (Toto et al., 2016), although measures were taken in a delimited region of interest centered on the foveal avascular zone.

As for the intermediate vascular plexus, the intraretinal vasculature in RP animals degenerated faster than the SP and slower than the DCP. In intermediate stages of retinal degeneration (P120), once the retina had already lost many photoreceptors, but before any changes were noticed in the inner retina, no signs of degeneration were found in the IP or SP. However, in advanced stages of degeneration (P480), once no photoreceptors remained and inner retinal remodeling was evident, the IP, like the DCP, had virtually disappeared. These results are in concordance with previous results in mouse models of RP, which showed that degeneration of the intermediate vascular plexus has a slower progression than that observed in the DCP (Otani et al., 2004). They also agree with studies reporting that horizontal and amacrine cells are required for the maintenance of the intermediate vascular plexus, and that dysfunction of these cells induces alterations in the intermediate vasculature (Usui et al., 2015).

Oxygen supply has been shown to be critical for the development and maintenance of the retinal architecture, and defects in the delicate balance between availability and consumption have been associated with a variety of retinal diseases (reviewed in Cuenca et al., 2014). In this sense, it has been reported that hyperoxia accelerates retinal degeneration, which may suggest that the increase in oxygen is a potential cause of retinal cell death (Stone et al., 1999; Shen et al., 2005), and that retinal hyperoxia contributes to retinal vessel atrophy (Penn et al., 2000). In RP disease, photoreceptor loss leads to abnormally high oxygen levels in the outer retina, whereas no anomalies are found in the superficial retina, IPL or choroid (Yu and Cringle, 2005). In agreement with this, our results show an early and rapid degeneration in the deeper retinal vessels, an intermediate rate of degeneration in the intermediate retinal vasculature, and a slight delayed dystrophy of the superficial vascular network. These results therefore suggest that photoreceptor death is the trigger of retinal vascular degeneration in RP. However, on the same token, the disruption of capillary plexuses and the loss of capillary loops may be altering the normal oxygen and nutrient supply to retinal cells, thereby accelerating the progress of retinal degeneration.

Retinal remodeling as a consequence of photoreceptor cell loss is widely accepted and well described by many authors (Jones et al., 2003, 2012; Marc et al., 2003; Cuenca et al., 2014; Pinilla et al., 2016). However, the mechanism through which photoreceptor degeneration is able to trigger alterations in the inner vascular plexus is still unknown. Vascular alterations in the superficial plexus have been described to be concomitant with astrogliosis and Müller cell activation in this animal model of retinal degeneration (Fernández-Sánchez et al., 2015a). Previous studies have revealed that astrocyte-derived VEGF acts as an endothelial cell survival factor (Alon et al., 1995; Fruttiger et al., 1996), and regulates in a dose-dependent manner vessel stabilization in the retinal vasculature (Scott et al., 2010; Groppa et al., 2015). Therefore, disease-associated changes in the functional status of retinal astrocytes may disrupt mechanisms involved in the maintenance of the superficial retinal vessels. Reduced expression of VEGF could be the basis of the progressive reduction of both capillary vessel and capillary loops in the DCP.

We can conclude that the death of photoreceptors is accompanied by degenerative changes in the retinal vascular network, leading to asymmetric remodeling of the three vascular plexuses. The atrophy of blood vessels and capillary loops is thought to affect the supply of oxygen and nutrients to retinal cells, thus speeding up the progression of retinal degeneration. Therefore, changes in the retinal vasculature must be taken into consideration when designing strategies for retinal protection and repair in patients with retinal degeneration.

## Author Contributions

NC: conception and design of the work. LF-S, GE and PL: data acquisition and analysis. LF-S, PL, IP and NC: data interpretation. LF-S, PL and NC: drafting of the manuscript.

## Conflict of Interest Statement

The authors declare that the research was conducted in the absence of any commercial or financial relationships that could be construed as a potential conflict of interest.

## References

[B1] Alon T. Hemo I. Itin A. Pe’er J. Stone J. Keshet E. (1995). Vascular endothelial growth factor acts as a survival factor for newly formed retinal vessels and has implications for retinopathy of prematurity. Nat. Med. 1, 1024–1028. 10.1038/nm1095-1024 7489357

[B2] Arend O. Wolf S. Jung F. Bertram B. Pöstgens H. Toonen H.. (1991). Retinal microcirculation in patients with diabetes mellitus: dynamic and morphological analysis of perifoveal capillary network. Br. J. Ophthalmol. 75, 514–518. 10.1136/bjo.75.9.514 1911651 PMC1042463

[B3] Battaglia Parodi M. Cicinelli M. V. Rabiolo A. Pierro L. Gagliardi M. Bolognesi G.. (2017). Vessel density analysis in patients with retinitis pigmentosa by means of optical coherence tomography angiography. Br. J. Ophthalmol. 101, 425–432. 10.1136/bjophthalmol-2016-308925 27343210

[B4] Battu R. Mohan A. Khanna A. Kumar A. Shetty R. (2015). Retinal oxygen saturation in retinitis pigmentosa and macular dystrophies in asian-indian eyes. Invest. Ophthalmol. Vis. Sci. 56, 2798–2802. 10.1167/iovs.14-15993 26024070

[B5] Bringmann A. Pannicke T. Grosche J. Francke M. Wiedemann P. Skatchkov S. N.. (2006). Müller cells in the healthy and diseased retina. Prog. Retin. Eye Res. 25, 397–424. 10.1016/j.preteyeres.2006.05.003 16839797

[B6] Chan G. Balaratnasingam C. Yu P. K. Morgan W. H. McAllister I. L. Cringle S. J.. (2012). Quantitative morphometry of perifoveal capillary networks in the human retina. Invest Ophthalmol Vis Sci. 53, 5502–5514. 10.1167/iovs.12-10265 22815351

[B7] Coorey N. J. Shen W. Chung S. (2012). The role of glia in retinal vascular disease. Clin. Exp. Optom. 95, 266–281. 10.1111/j.1444-0938.2012.00741.x 22519424

[B8] Cuenca N. Fernández-Sánchez L. Campello L. Maneu V. De la Villa P. Lax P.. (2014). Cellular responses following retinal injuries and therapeutic approaches for neurodegenerative diseases. Prog. Retin. Eye Res. 43, 17–75. 10.1016/j.preteyeres.2014.07.001 25038518

[B9] Cuenca N. Pinilla I. Sauvé Y. Lu B. Wang S. Lund R. D. (2004). Regressive and reactive changes in the connectivity patterns of rod and cone pathways of P23H transgenic rat retina. Neuroscience 127, 301–317. 10.1016/j.neuroscience.2004.04.042 15262321

[B10] Dorrell M. I. Aguilar E. Friedlander M. (2002). Retinal vascular development is mediated by endothelial filopodia, a preexisting astrocytic template and specific R-cadherin adhesion. Invest. Ophthalmol. Vis. Sci. 43, 3500–3510. 12407162

[B11] Dryja T. P. McEvoy J. A. McGee T. L. Berson E. L. (2000). Novel rhodopsin mutations Gly114Val and Gln184Pro in dominant retinitis pigmentosa. Invest. Ophthalmol. Vis. Sci. 41, 3124–3127. 10967073

[B12] Esquiva G. Lax P. Cuenca N. (2013). Impairment of intrinsically photosensitive retinal ganglion cells associated with late stages of retinal degeneration. Invest. Ophthalmol. Vis. Sci. 54, 4605–4618. 10.1167/iovs.13-12120 23766478

[B13] Esquiva G. Lax P. Pérez-Santonja J. J. García-Fernández J. M. Cuenca N. (2017). Loss of melanopsin-expressing ganglion cell subtypes and dendritic degeneration in the aging human retina. Front. Aging Neurosci. 9:79. 10.3389/fnagi.2017.00079 28420980 PMC5378720

[B14] Eysteinsson T. Hardarson S. H. Bragason D. Stefánsson E. (2014). Retinal vessel oxygen saturation and vessel diameter in retinitis pigmentosa. Acta Ophthalmol. 92, 449–453. 10.1111/aos.12359 24767302

[B15] Fernández-Sánchez L. Bravo-Osuna I. Lax P. Arranz-Romera A. Maneu V. Esteban-Pérez S.. (2017). Controlled delivery of tauroursodeoxycholic acid from biodegradable microspheres slows retinal degeneration and vision loss in P23H rats. PLoS One 12:e0177998. 10.1371/journal.pone.0177998 28542454 PMC5444790

[B16] Fernández-Sánchez L. Lax P. Campello L. Pinilla I. Cuenca N. (2015a). Astrocytes and muller cell alterations during retinal degeneration in a transgenic rat model of retinitis pigmentosa. Front. Cell Neurosci. 9:484. 10.3389/fncel.2015.00484 26733810 PMC4686678

[B17] Fernández-Sánchez L. Lax P. Esquiva G. Martín-Nieto J. Pinilla I. Cuenca N. (2012a). Safranal, a saffron constituent, attenuates retinal degeneration in P23H rats. PLoS One 7:e43074. 10.1371/journal.pone.0043074 22900092 PMC3416780

[B18] Fernández-Sánchez L. Lax P. Isiegas C. Ayuso E. Ruiz J. M. de la Villa P.. (2012b). Proinsulin slows retinal degeneration and vision loss in the P23H rat model of retinitis pigmentosa. Hum Gene Ther. 23, 1290–1300. 10.1089/hum.2012.067 23017108 PMC3523255

[B19] Fernández-Sánchez L. Lax P. Noailles A. Angulo A. Maneu V. Cuenca N. (2015b). Natural compounds from saffron and bear bile prevent vision loss and retinal degeneration. Molecules 20, 13875–13893. 10.3390/molecules200813875 26263962 PMC6332441

[B20] Fernández-Sánchez L. Lax P. Pinilla I. Martín-Nieto J. Cuenca N. (2011). Tauroursodeoxycholic acid prevents retinal degeneration in transgenic P23H rats. Invest. Ophthalmol. Vis. Sci. 52, 4998–5008. 10.1167/iovs.11-7496 21508111

[B21] Flynn J. T. Chan-Ling T. (2006). Retinopathy of prematurity: two distinct mechanisms that underlie zone 1 and zone 2 disease. Am. J. Ophthalmol. 142, 46–59. 10.1016/j.ajo.2006.02.018 16815250

[B22] Fruttiger M. Calver A. R. Krüger W. H. Mudhar H. S. Michalovich D. Takakura N.. (1996). PDGF mediates a neuron-astrocyte interaction in the developing retina. Neuron 17, 1117–1131. 10.1016/s0896-6273(00)80244-5 8982160

[B23] Garcìa-Ayuso D. Di Pierdomenico J. Esquiva G. Nadal-Nicolás F. M. Pinilla I. Cuenca N.. (2015). Inherited photoreceptor degeneration causes the death of melanopsin-positive retinal ganglion cells and increases their coexpression of brn3a. Investig. Ophthalmol. Vis. Sci. 56, 4592–4604. 10.1167/iovs.15-16808 26200499

[B24] García-Ayuso D. Salinas-Navarro M. Agudo M. Cuenca N. Pinilla I. Vidal-Sanz M.. (2010). Retinal ganglion cell numbers and delayed retinal ganglion cell death in the P23H rat retina. Exp. Eye Res. 91, 800–810. 10.1016/j.exer.2010.10.003 20955700

[B25] Garcia-Ayuso D. Salinas-navarro M. Agudo-barriuso M. Alarcón-martínez L. Vidal-sanz M. Villegas-pérez M. P. (2011). Retinal ganglion cell axonal compression by retinal vessels in light- induced retinal degeneration. Mol. Vis. 17, 1716–1733. 21738401 PMC3130728

[B26] Gariano R. F. Gardner T. W. (2005). Retinal angiogenesis in development and disease. Nature 438, 960–966. 10.1038/nature04482 16355161

[B27] Groppa E. Brkic S. Bovo E. Reginato S. Sacchi V. Di Maggio N.. (2015). VEGF dose regulates vascular stabilization through Semaphorin3A and the Neuropilin-1+ monocyte/TGF-β1 paracrine axis. EMBO Mol. Med. 7, 1366–1384. 10.15252/emmm.201405003 26323572 PMC4604689

[B28] Hartong D. T. Berson E. L. Dryja T. P. (2006). Retinitis pigmentosa. Lancet 368, 1795–1809. 10.1016/S0140-6736(06)69740-7 17113430

[B29] Haverkamp S. Kolb H. Cuenca N. (2000). Morphological and neurochemical diversity of neuronal nitric oxide synthase-positive amacrine cells in the turtle retina. Cell Tissue Res. 302, 11–19. 10.1007/s004410000267 11079711

[B30] Hughes S. Gardiner T. Hu P. Baxter L. Rosinova E. Chan-Ling T. (2006). Altered pericyte-endothelial relations in the rat retina during aging: Implications for vessel stability. Neurobiol. Aging 27, 1838–1847. 10.1016/j.neurobiolaging.2005.10.021 16387390

[B31] Jones B. W. Pfeiffer R. L. Ferrell W. D. Watt C. B. Marmor M. Marc R. E. (2016). Retinal remodeling in human retinitis pigmentosa. Exp. Eye Res. 150, 149–165. 10.1016/j.exer.2016.03.018 27020758 PMC5031517

[B32] Jones B. W. Watt C. B. Frederick J. M. Baehr W. Chen C.-K. Levine E. M.. (2003). Retinal remodeling triggered by photoreceptor degenerations. J. Comp. Neurol. 464, 1–16. 10.1002/cne.10703 12866125

[B33] Jones B. W. Kondo M. Terasaki H. Lin Y. McCall M. Marc R. E. (2012). Retinal remodeling. Jpn. J. Ophthalmol. 56, 289–306. 10.1007/s10384-012-0147-2 22644448 PMC3726038

[B34] Kaur C. Foulds W. S. Ling E. A. (2008). Blood-retinal barrier in hypoxic ischaemic conditions: basic concepts, clinical features and management. Prog. Retin. Eye Res. 27, 622–647. 10.1016/j.preteyeres.2008.09.003 18940262

[B35] Kolomiets B. Dubus E. Simonutti M. Rosolen S. Sahel J. A. Picaud S. (2010). Late histological and functional changes in the P23H rat retina after photoreceptor loss. Neurobiol. Dis. 38, 47–58. 10.1016/j.nbd.2009.12.025 20060471

[B36] Kur J. Newman E. A. Chan-Ling T. (2012). Cellular and physiological mechanisms underlying blood flow regulation in the retina and choroid in health and disease. Prog. Retin. Eye Res. 31, 377–406. 10.1016/j.preteyeres.2012.04.004 22580107 PMC3418965

[B37] Lax P. Esquiva G. Altavilla C. Cuenca N. (2014). Neuroprotective effects of the cannabinoid agonist HU210 on retinal degeneration. Exp. Eye Res. 120, 175–185. 10.1016/j.exer.2014.01.019 24495949

[B38] Lax P. Esquiva G. Fuentes-Broto L. Segura F. Sánchez-Cano A. Cuenca N.. (2016). Age-related changes in photosensitive melanopsin-expressing retinal ganglion cells correlate with circadian rhythm impairments in sighted and blind rats. Chronobiol. Int. 33, 374–391. 10.3109/07420528.2016.1151025 27003747

[B39] Lax P. Otalora B. B. Esquiva G. Rol M. L. Madrid J. A. Cuenca N. (2011). Circadian dysfunction in P23H rhodopsin transgenic rats: effects of exogenous melatonin. J Pineal Res. 50, 183–191. 10.1111/j.1600-079X.2010.00827.x 21062354

[B40] Liu H. Tang J. Du Y. Saadane A. Tonade D. Samuels I.. (2016). Photoreceptor cells influence retinal vascular and diabetes. Invest. Ophthalmol. Vis. Sci. 57, 4272–4281. 10.1167/iovs.16-19415 27548901 PMC5015983

[B41] Lopez Torres L. T. Türksever C. Schötzau A. Orgül S. Todorova M. G. (2015). Peripapillary retinal vessel diameter correlates with mfERG alterations in retinitis pigmentosa. Acta Ophthalmol. 93, e527–e533. 10.1111/aos.12707 25809154

[B42] Machida S. Kondo M. Jamison J. A. Khan N. W. Kononen L. T. Sugawara T.. (2000). P23H rhodopsin transgenic rat: correlation of retinal function with histopathology. Invest. Ophthalmol. Vis. Sci. 41, 3200–3209. 10967084

[B43] Marc R. E. Jones B. W. Watt C. B. Strettoi E. (2003). Neural remodeling in retinal degeneration. Prog. Retin. Eye Res. 22, 607–655. 10.1016/s1350-9462(03)00039-9 12892644

[B44] Milam A. H. Li Z. Y. Fariss R. N. (1998a). Histopathology of the human retina in retinitis pigmentosa. Prog. Retin. Eye Res. 17, 175–205. 10.1016/S1350-9462(97)00012-8 9695792

[B45] Milam A. H. Li Z. Fariss R. N. (1998b). Histology of the human retina in retinitis pigmentosa. Prog. Retin. Eye Res. 17, 175–205. 10.1016/S1350-9462(97)00012-8 9695792

[B46] Newsome D. A. (1986). Retinal fluorescein leakage in retinitis pigmentosa. Am. J. Ophthalmol. 101, 354–360. 10.1016/0002-9394(86)90831-7 3953729

[B47] Noailles A. Fernández-Sánchez L. Lax P. Cuenca N. (2014). Microglia activation in a model of retinal degeneration and TUDCA neuroprotective effects. J. Neuroinflammation 11:186. 10.1186/s12974-014-0186-3 25359524 PMC4221719

[B48] Noailles A. Maneu V. Campello L. Gómez-Vicente V. Lax P. Cuenca N. (2016). Persistent inflammatory state after photoreceptor loss in an animal model of retinal degeneration. Sci. Rep. 6:33356. 10.1038/srep33356 27624537 PMC5022039

[B49] Noailles A. Maneu V. Campello L. Lax P. Cuenca N. (2018). Systemic inflammation induced by lipopolysaccharide aggravates inherited retinal dystrophy. Cell Death Dis. 9:350. 10.1038/s41419-018-0355-x 29500424 PMC5834451

[B50] Otani A. Dorrell M. I. Kinder K. Moreno S. K. Nusinowitz S. Banin E.. (2004). Rescue of retinal degeneration by intravitreally injected adult bone marrow-derived lineage-negative hematopoietic stem cells. J. Clin. Invest. 114, 765–774. 10.1172/JCI21686 15372100 PMC516263

[B51] Penn J. S. Li S. Naash M. I. (2000). Ambient hypoxia reverses retinal vascular attenuation in a transgenic mouse model of autosomal dominant retinitis pigmentosa. Invest. Ophthalmol. Vis. Sci. 41, 4007–4013. 11053306

[B52] Penn J. S. Madan A. Caldwell R. B. Bartoli M. Caldwell R. W. Hartnett M. E. (2008). Vascular endothelial growth factor in eye disease. Prog. Retin. Eye Res. 27, 331–371. 10.1016/j.preteyeres.2008.05.001 18653375 PMC3682685

[B53] Pennesi M. E. Nishikawa S. Matthes M. T. Yasumura D. LaVail M. M. (2008). The relationship of photoreceptor degeneration to retinal vascular development and loss in mutant rhodopsin transgenic and RCS rats. Exp. Eye Res. 87, 561–570. 10.1016/j.exer.2008.09.004 18848932 PMC3541029

[B54] Pinilla I. Fernández-Sánchez L. Segura F. J. Sánchez-Cano A. I. Tamarit J. M. Fuentes-Broto L.. (2016). Long time remodeling during retinal degeneration evaluated by optical coherence tomography, immunocytochemistry and fundus autofluorescence. Exp. Eye Res. 150, 122–134. 10.1016/j.exer.2015.10.012 26521765

[B55] Pournaras C. J. Rungger-Brändle E. Riva C. E. Hardarson S. H. Stefansson E. (2008). Regulation of retinal blood flow in health and disease. Prog. Retin. Eye Res. 27, 284–330. 10.1016/j.preteyeres.2008.02.002 18448380

[B56] Provis J. M. (2001). Development of the primate retinal vasculature. Prog. Retin. Eye Res. 20, 799–821. 10.1016/s1350-9462(01)00012-x 11587918

[B57] Puthussery T. Taylor W. R. (2010). Functional changes in inner retinal neurons in animal models of photoreceptor degeneration. Adv. Exp. Med. Biol. 664, 525–532. 10.1007/978-1-4419-1399-9_60 20238055

[B58] Scott A. Powner M. B. Gandhi P. Clarkin C. Gutmann D. H. Johnson R. S.. (2010). Astrocyte-derived vascular endothelial growth factor stabilizes vessels in the developing retinal vasculature. PLoS One 5:e11863. 10.1371/journal.pone.0011863 20686684 PMC2912336

[B59] Semkova I. Kreppel F. Welsandt G. Luther T. Kozlowski J. Janicki H.. (2002). Autologous transplantation of genetically modified iris pigment epithelial cells: a promising concept for the treatment of age-related macular degeneration and other disorders of the eye. Proc. Natl. Acad. Sci. U S A 99, 13090–13095. 10.1073/pnas.202486199 12239351 PMC130591

[B60] Shen J. Yang X. Dong A. Petters R. M. Peng Y. W. Wong F.. (2005). Oxidative damage is a potential cause of cone cell death in retinitis pigmentosa. J. Cell. Physiol. 203, 457–464. 10.1002/jcp.20346 15744744

[B61] Steinberg R. H. Flannery J. G:. Naash M. Oh P. Matthes M. T. Yasumura D. (1996). Transgenic rat models of inherited retinal degeneration caused by mutant opsin genes. Investig. Ophthalmol. Vis. Sci. 37:S698.

[B62] Stone J. Itin A. Alon T. Pe’er J. Gnessin H. Chan-Ling T.. (1995). Development of retinal vasculature is mediated by hypoxia-induced vascular endothelial growth factor (VEGF) expression by neuroglia. J. Neurosci. 15, 4738–4747. 10.1523/JNEUROSCI.15-07-04738.1995 7623107 PMC6577882

[B63] Stone J. Maslim J. Valter-Kocsi K. Mervin K. Bowers F. Chu Y.. (1999). Mechanisms of photoreceptor death and survival in mammalian retina. Prog. Retin. Eye Res. 18, 689–735. 10.1016/s1350-9462(98)00032-9 10530749

[B64] Toto L. Borrelli E. Mastropasqua R. Senatore A. Di Antonio L. Di Nicola M.. (2016). Macular features in retinitis pigmentosa: correlations among ganglion cell complex thickness, capillary density and macular function. Investig. Opthalmology Vis. Sci. 57, 6360–6366. 10.1167/iovs.16-20544 27898981

[B65] Türksever C. Valmaggia C. Orgül S. Schorderet D. F. Flammer J. Todorova M. (2014). Retinal vessel oxygen saturation and its correlation with structural changes in retinitis pigmentosa. Acta Ophthalmol. 92, 454–460. 10.1111/aos.12379 24767408

[B66] Usui Y. Westenskow P. D. Kurihara T. Aguilar E. Sakimoto S. Paris L. P.. (2015). Neurovascular crosstalk between interneurons and capillaries is required for vision. J. Clin. Invest. 125, 2335–2346. 10.1172/JCI80297 25915585 PMC4497761

[B67] Villegas-Pérez M. P. Lawrence J. M. Vidal-Sanz M. Lavail M. M. Lund R. D. (1998). Ganglion cell loss in RCS rat retina: A result of compression of axons by contracting intraretinal vessels linked to the pigment epithelium. J. Comp. Neurol. 392, 58–77. 10.1002/(sici)1096-9861(19980302)392:1<58::AID-CNE5>3.3.CO;2-O 9482233

[B68] Wang S. Paz M. Villegas-Pérez M. P. Vidal-Sanz M. Lund R. D. (2000). Progressive Optic axon dystrophy and vascular changes in rd mice. Invest. Ophthalmol. Vis. Sci. 41, 537–545. 10670486

[B69] Wang S. Villegas-Pérez M. P. Holmes T. Lawrence J. M. Vidal-Sanz M. Hurtado-Montalbán N.. (2003). Evolving neurovascular relationships in the RCS rat with age. Curr. Eye Res. 27, 183–196. 10.1076/ceyr.27.3.183.16053 14562184

[B100] Yu D. Y. Cringle S. J. (2001). Oxygen distribution and consumption within the retina in vascularised and avascular retinas and in animal models of retinal disease. Prog. Retin. Eye Res. 20, 175–208. 10.1016/S1350-9462(00)00027-6 11173251

[B70] Yu D. Y. Cringle S. J. (2005). Retinal degeneration and local oxygen metabolism. Exp. Eye Res. 80, 745–751. 10.1016/j.exer.2005.01.018 15939030

[B71] Yu D. Y. Cringle S. J. Su E. N. Yu P. K. (2000). Intraretinal oxygen levels before and after photoreceptor loss in the RCS rat. Invest. Ophthalmol. Vis. Sci. 41, 3999–4006. 11053305

